# Responses on Must and Wine Composition of *Vitis vinifera* L. cvs. Riesling and Cabernet Sauvignon under a Free Air CO_2_ Enrichment (FACE)

**DOI:** 10.3390/foods10010145

**Published:** 2021-01-12

**Authors:** Yvette Wohlfahrt, Claus-Dieter Patz, Dominik Schmidt, Doris Rauhut, Bernd Honermeier, Manfred Stoll

**Affiliations:** 1 Department of General and Organic Viticulture, Hochschule Geisenheim University, Von-Lade-Strasse 1, 65366 Geisenheim, Germany; manfred.stoll@hs-gm.de; 2 Department of Beverage Research, Hochschule Geisenheim University, Von-Lade-Strasse 1, 65366 Geisenheim, Germany; claus.patz@hs-gm.de; 3 Department of Modeling and Systems Analysis, Hochschule Geisenheim University, Von-Lade-Strasse 1, 65366 Geisenheim, Germany; dominik.schmidt@hs-gm.de; 4 Department of Microbiology and Biochemistry, Hochschule Geisenheim University, Von-Lade-Strasse 1, 65366 Geisenheim, Germany; doris.rauhut@hs-gm.de; 5 Department of Agronomy and Plant Breeding I, Justus Liebig University, Schubertstrasse 81, 35392 Giessen, Germany; bernd.honermeier@agrar.uni-giessen.de

**Keywords:** FACE, CO_2_ enrichment, climate change, *Vitis vinifera*, must, wine analysis, composition, anthocyanins, monomeric index, colorimetric parameters

## Abstract

Challenges of climate change on the future grape and wine production are widely discussed in science and in the wine industry with the goal to maintain a consistent must and wine quality in the future. Therefore, the effect of elevated CO_2_ (eCO_2_)—as one of the relevant greenhouse gases jointly responsible for a changing climate—was investigated concerning the composition of must and wine made of two grapevine cultivars *V. vinifera* L. cvs. Riesling and Cabernet Sauvignon within the established VineyardFACE (Free-Air Carbon dioxide Enrichment) experiment. Must and wine analysis were conducted in three consecutive years (2014–2016) by analyzing standard must and wine parameters, e.g., total soluble solids (TSS), pH, total acidity (TA), organic acids (e.g., tartaric acid, malic acid, shikimic acid, citric acid, volatile acid and gluconic acid) or total phenolics (TP). Also, for both cultivars CIELab coordinates (L* for lightness, a* as green/red and b* as blue/yellow components) were used to test colour in young white and red wines. Additionally, total anthocyanins and monomeric indices were analyzed for young wines of the red cultivar Cabernet Sauvignon. With marginal differences between CO_2_ treatments, the composition of must and young wines was not found to be negatively influenced by an eCO_2_ concentration.

## 1. Introduction

One of the most relevant greenhouse gases, atmospheric carbon dioxide (CO_2_), has been increasing continuously since pre-industrial times. The Intergovernmental Panel on Climate Change (IPCC) has predicted an average atmospheric CO_2_-increase of 2.25 ppm per year based on four main emission-scenarios [1]. Compared to the current CO_2_ concentration this will result in an increase of about 20% by the mid-21st century up to 550 ppm in atmospheric CO_2_ concentration. The closely linked increase in global mean surface temperature together with elevated CO_2_ concentrations, leads to a potential alteration in plant physiology, yield performance and fruit quality of perennial crops. Grapevines as special crop plants are widely recognized for being sensitive to climate change, and numerous studies have been investigated over the last decades dealing with grapevine physiology, yield efficiency and grape and wine composition responses to changing environmental conditions [2,3,4,5,6,7,8,9,10,11].

Notably, CO_2_ enrichment field studies using open top chambers [12,13,14,15], Mini-FACE [16,17,18] or the recently reported VineyardFACE system [19,20,21] investigated the effects of elevated CO_2_ on grapevine response. Main results of these studies showed increased net assimilation rates and therefore an increase in vigour and yield parameters of vines without negatively affecting fruit or wine quality. It was discussed if due to increased berry weights of *V. vinifera* under eCO_2_ conditions [13,16,19,21,22] the grape and wine quality might be affected, as it seems that berry size is associated to fruit and wine composition [22,23,24,25,26,27,28,29]. According to previous studies, grapevines exposed to eCO_2_ concentrations showed an alteration in total soluble solid accumulation and acid degradation over the period of berry development in general [12,15,17,21,30]. Within a recent study using the VineyardFACE experiment, it was shown that eCO_2_ resulted in enhanced berry weights and higher malic acid for both cultivars in two years and lower tartaric acid for Riesling in one year at the end of berry ripening, while sugar accumulation remained unchanged [21]. Total soluble solids of must at harvest were not influenced by eCO_2_ [12,17,19]. Only few studies [12,17] observed that accumulation of total soluble solids in berries of red cultivars under Mediterranean climate increased under eCO_2_ during ripening of berries, but at harvest the effect disappeared. Only Australian Shiraz accumulated higher total soluble solids at harvest under eCO_2_ over years compared to control vines [15].

Besides sugar accumulation and acid degradation, the concentration of anthocyanins in red grape cultivars grown under a warmer climate is described to potentially increase under eCO_2_ concentration [5,17,31]. A coherency of the annual atmospheric rise of CO_2_ and the total anthocyanin concentration was reported in an Australian study, where vintage composition of ten years was linked to abiotic parameters [31]. Chemical analysis in red wines of cv. Sangiovese showed higher amounts of total flavonoids, total anthocyanins and non-anthocyanin flavonoids under eCO_2_ in one out of two vintages, even though values were higher in both years [17]. Authors assumed that under a lower CO_2_ enrichment level (550 ppm) pigments tended to be more stimulated than under higher eCO_2_ level (700 ppm). No differences between CO_2_ treatments were detected for total polyphenols, colour intensity, colour tonality, alcohol, total and volatile acidity or pH of red wines [17]. Gonçalves et al. [12] reported similar results for the red cultivar Touriga Franca and the parameters total and volatile acidity as well as antioxidant capacity. The opposite was found in anthocyanin concentration and polyphenols, which were inhibited under eCO_2_ conditions. In addition, higher alcohol and lower pH were found under eCO_2_ in one year, whereas density decreased for eCO_2_ treatment in the year after [12]. Overall, authors embraced that the effect of eCO_2_ on wine composition under Mediterranean climate did not affect wine quality at the end [12,17].

The aim of this study was to investigate if eCO_2_ affects must and wine composition of two cultivars (*V. vinifera* L. cvs. Riesling and Cabernet Sauvignon) over three consecutive years under a temperate oceanic climate by analyzing standard must and wine and additional colorimetric parameters.

## 2. Materials and Methods

### 2.1. Field Site, Experimental Design and Plant Material

The experimental study was performed at the VineyardFACE field site (49° 59′ N, 7° 57′ E) located at Hochschule Geisenheim University in the Rheingau Valley, Germany. The VineyardFACE field trial was established in 2013 as a ring-shaped system and an area of about 0.5 hectares. The training system of vines was a vertical shoot positioning system (VSP) and canes were pruned to five nodes m^−2^. The planting distance of vines was 0.9 m × 1.8 m and rows were north-south orientated. The cultivars *Vitis vinifera* L. cv. Riesling (clone 198–30 Gm) grafted on rootstock SO4 (clone 47 Gm) and cv. Cabernet Sauvignon (clone 170) grafted on rootstock 161–49 Couderc were used.

The VineyardFACE experiment was established with two CO_2_ treatments, ambient (aCO_2_, 400 ppm) and elevated (eCO_2_, +20% of the aCO_2_ treatment). Both CO_2_ treatments were replicated three times with aCO_2_ rings as A1, A2 and A3, while eCO_2_ rings were E1, E2 and E3. Each ring contained seven rows of vines, which were planted with Riesling and Cabernet Sauvignon. Vines in eCO_2_ rings were fumigated with +20% of current atmospheric CO_2_ from sunrise to sunset over the three years of the experiment, while aCO_2_ rings were operated under atmospheric CO_2_ conditions. Only the five inner rows of the rings were used for data collection with 23 vines per ring of Riesling and 24 vines per ring of Cabernet Sauvignon. A detailed description of the VineyardFACE field trial and the function of CO_2_ fumigation and distribution was presented previously [19].

### 2.2. Weather Conditions

**Figure 1:**
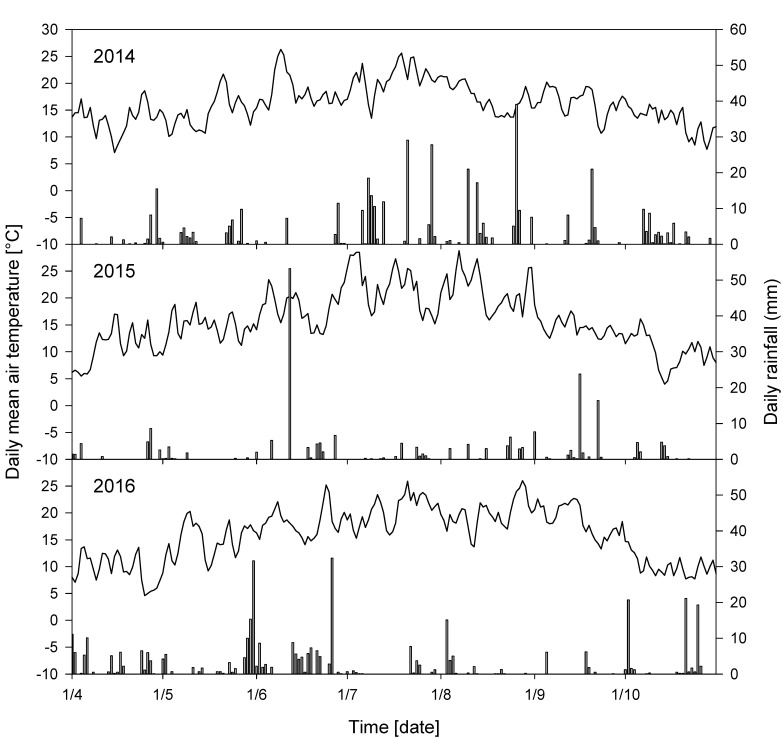
Mean air temperature (solid line) and daily rainfall (black bars) during vegetation periods (1 April to 31 October) 2014, 2015 and 2016 at the Geisenheim VineyardFACE.

The climate in Geisenheim, Rheingau is described as a temperate oceanic climate with warm summers and mild winters. The average annual air temperature is 10.5 °C (long-term average from 1981–2010) and mean annual rainfall is 543 mm. Weather data were collected from a weather station located at the VineyardFACE site. Daily rainfall and daily mean air temperature for the vegetation periods (1 April to 31 October) 2014, 2015 and 2016 are shown in Figure 1. Average temperatures during vegetation periods were 16.3 °C in 2014, 15.9 °C in 2015 and 15.9 °C in 2016. Total precipitation during vegetation periods was 441 mm, 227 mm and 371 mm, respectively.

### 2.3. Experimental Winemaking

At harvest date, handpicked grapes of 23 *V. vinifera* Riesling vines per ring were processed as microvinifications using a standardized white microvinification protocol. For each single microvinification, approximately 50 kg of grapes were crushed, pressed and transferred to 30 L glass carboys. To the obtained grape juice 50 mg/L SO_2_ (potassium bisulphite solution) were added. Carboys were stored overnight at 5 °C in a cold store. After 12 h clear juice was racked into 30 L carboys and inoculated with 20 g/hL dry yeast culture LALVIN^®^ EC-1118 (Eaton, Langenlonsheim, Germany). After finishing fermentation to dryness, wines were racked off the lees, sulfured with 100 mg/L SO_2_ and transferred into 25 L carboys. Wines were stored at cellar temperature at 15 °C until bottling. Six months later, wines were filtrated using K250 filter sheets (Seitz K Series, Pall Food and Beverage). Afterwards wines were bottled, adding 50 mg/L free SO_2_ after cartridge filtration (0.45 µm). For bottling, 0.5 L bottles with screw cap closures were used.

For Cabernet Sauvignon handpicked grapes of 24 vines per ring were processed as microvinifications using a standardized red microvinification protocol with fermentation on grape skins. For each single microvinification, approximately 40 kg of grapes were destemmed, crushed and transferred into 60 L plastic vessels (Speidel). To the obtained grape mash 50 mg/L SO_2_ were added. Two hours later grape mash was inoculated with 20 g/hL SIHA^®^ Active Yeast 8 (Eaton, Langenlonsheim, Germany). During fermentation, mash was plunged twice a day. In 2014, 3 days after inoculation the fermenting mash was chaptalized with 40 g/L saccharose to reach an appropriate alcohol level at the end of fermentation. After fermentation was completed, the grape mash was pressed, transferred into 30 L carboys and stored overnight at 5 °C in a cold store. Clear wine was racked into 30 L carboys after 12 h of sedimentation. Three days later, wines were inoculated with 10 g/hL of a malolactic fermentation culture Biostart^®^ Vitale Sk 11^®^ (Erbslöh, Geisenheim, Germany). When malolactic fermentation was completed wines were racked of the fine lees and 80 mg/L SO_2_ was added. Five months later, wines were filtrated using K250 filter sheets (Seitz K Series, Pall Food and Beverage). Wines were bottled with 50 mg/L free SO_2_ after cartridge filtration (0.45 µm) using 0.5 L bottles with screw cap closures.

### 2.4. Grape Must Sampling

Must at harvest was obtained directly after pressing Riesling grape batches and samples were collected in 50 mL tubes. Cabernet Sauvignon must was sampled from grape mash batches directly after crushing and were also collected in 50 mL tubes. Must samples were centrifuged (5430R, Eppendorf AG, Hamburg, Germany) for 5 min at 7830 rpm and 20 °C. By using a handheld refractometer (HRKL32, Krüss, Hamburg, Germany) TSS (°Brix) of samples was analyzed.

### 2.5. High-Performance Liquid Chromatography (HPLC) Analysis of Organic Acids and Monosaccharides

Analysis of monosaccharides (fructose and glucose) and organic acids (tartaric, malic, citric and shikimic acid) were conducted using high performance liquid chromatography (HPLC) described by Schneider et al. [32] and modified by Knoll et al. [33]. The following changes were made: 5 μL of sample was injected into the Agilent Technologies 1100 series liquid chromatograph equipped with a multiwave-length detector (MWD) and analyzed using an Allure^®^ Organic Acid column (250 mm × 4.6 mm inside diameter) (Restek GmbH, Bad Homburg, Germany) with a Security Guard^TM^ Cartridge C18 4 × 3 mm (Phenomenex, Aschaffenburg, Germany). As an eluent, purified water was used with 0.0139% sulfuric acid and 0.5% (*v*/*v*) ethanol. The column was operated at 46 °C with an eluent flow rate at 0.6 mL/min. Eluting compounds were detected by UV absorbance at 210 nm.

### 2.6. FT-MIR and NMR Analysis

Standard must and wine parameters were measured via liquid Fourier transform-middle infrared spectrometry (FT-MIR) using a FOSS WineScan FT120 FT-MIR spectrometer equipped with a DTGS pyroelectric detector as described by Patz et al. [34]. Nuclear magnetic resonance analysis (NMR) was used to assess additional wine components, e.g., 2,3-butanediol, 2-phenylethanol or 3-methyl-butanol as shown in Table 1 [35]. NMR was performed under full automation using an AVANCE III 400 (Bruker BioSpin GmbH, Rheinstetten, Germany) equipped with a 5 mm 1H/D-TXI probe head with z-gradient, automated tuning and matching accessory, and BTO-2000 for temperature control. All spectra were processed in full automation using TOPSPIN 2.1 (Bruker BioSpin GmbH, Rheinstetten, Germany).

### 2.7. Preparation of Wine Samples

Unfiltered white and red wine samples were obtained after racking off the fine lees prior SO_2_ addition and were collected in 50 mL tubes. Tubes were centrifuged (Rotina 35, Hettich, Tuttlingen, Germany) at 12,850 rpm for 6 min at 20 °C.

### 2.8. Quantification of Total Phenols and Trolox Equivalent Antioxidative Capacity (TEAC)

Total phenolics were assayed with the Folin–Ciocalteu method based on a (+) catechin calibration [36]. Subsequent spectrophotometric analysis were conducted with a Konelab 20 Xti analyzer (Thermo Fisher, Dreieich, Germany). Antioxidant capacity was determined using Trolox equivalent antioxidative capacity (TEAC) and was expressed as Trolox equivalents in mM of Trolox per litre (mmol TEAC/L wine) as described earlier [37].

### 2.9. Colorimetric Parameters

The absorbance spectra of wines (380–770 nm, 2 nm step) were recorded for Cabernet Sauvignon (red wine) in 2 mm or rather for Riesling in 10 mm (white wine) width quartz cuvettes using a Unicam UV 500 spectrophotometer with a wolfram lamp and sipper (Thermo Spectronic, Dreieich, Germany) at Intelliscan speed (nm/min) using Vision pro software (Version 2.03). Results were calculated for 10 mm optical path length.

The absorbance spectra data were used to calculate the CIELab color coordinates following the standard method of the Commission Internationale de L’Eclairage (CIE, 1976), which is defined as OIV method OIV-MA-AS2-11 [38]. The CIELab coordinates L* (lightness), a* (green/red component), and b* (blue/yellow component) were calculated using the Red Wine Color Report (ETS Laboratories) by using CIE 10° standard observer and standard illuminant D65.

The monomeric index (MI) as a ratio of monomeric and polymeric anthocyanins of red wines was determined as described by Giusti and Wrolstad [39], and modified by Bonerz et al. [40]. The measurements for monomeric and polymeric anthocyanins were carried out with a spectrophotometer (UVmini 1240, Shimadzu, Suzhou, China) with λ = 520 nm.

### 2.10. High-Performance Liquid Chromatography (HPLC) Analysis of Anthocyanins

HPLC analysis of anthocyanins was carried out on a Dionex HPLC system equipped with a Dionex PDA-100 photodiode array detector (wavelength 260–650 nm) (Thermo Fisher, Dreieich, Germany) and a Dionex STH 585 column oven according to Würth et al. [41] and modified by Hey et al. [42]. Separation was performed on a reversed phase LiChrospher 100 RP-18 (250 mm × 3 mm, 5 µm, Merck, Darmstadt, Germany) at 20 °C. A gradient consisting of solvent A (water/acetonitrile/o-phosphoric acid (85%) (94/4/2, *v*/*v*/*v*) and solvent B (water/acetonitrile/o-phosphoric acid (85%) (48/50/2, *v*/*v*/*v*) was applied at a flow rate of 500 μL/min. An aliquot of 20 µL of red wine, previously filtered through a 0.45 μm RC syringe filter (Durafill, Duratec GmbH, Hockenheim, Germany), was injected onto the column. Quantification was carried out by peak area measurements at 520 nm. The standard report was performed using the chromatography software Dionex™ Chromeleon™ (Version 6.8, Thermo Scientific™, Dreieich, Germany). All analyses were operated in duplicate. The concentration of each anthocyanin was expressed as the equivalent of malvidin-3-*O*-glucoside.

### 2.11. Statistical Analysis

Inferential statistical analysis was performed using R, version 3.6.3 [43]. Bayesian generalized linear mixed model analyses (R-package brms, version 2.12.0) [44,45,46] were applied for each cultivar (Riesling, Cabernet Sauvignon) and all continuous numerical measures from must and wine analysis [21]. The model accounts for structure of sampling, pseudo replications (block, ring) and respective repeated measures in time (year). Fixed effects were estimated for treatment (aCO_2_, eCO_2_), year, and the treatment × year interaction. Years were treated as factorial variables as confounded effects, e.g., temperature, precipitation, etc., were not considered. Inference was focused on the estimation of differences between aCO_2_ and eCO_2_.

A Gamma distribution likelihood with a log-link function was supposed for all measures, except L*a*b* measurements, to account for deviations from normality and the certainty that all measures are confined to positive values only, while assuming the variance to increase with the mean, which is typical for ecological data [47]. Analysis on L*a*b* measures use a Gaussian distribution likelihood. Weakly informative priors were set for the intercept and effect sizes. Models were run using four Markov chains with a warm-up phase of 4000 iterations, followed by 4000 samplings iterations per chain. Hence, each posterior consisted of a total of 16,000 samples. To estimate the difference between treatments posterior predictions for each year were used. Results included the probability (%) of the treatment effect being larger than zero by estimating the proportion of posterior predicted differences between eCO_2_ and aCO_2_ that was greater than zero. A significant difference was attested when the probability of the treatment effect was estimated to be above 90% (positive difference) or below 10% (negative difference).

This restrictive threshold was chosen to reliably detected consistent differences, as higher residual errors, causing higher uncertainties in posterior predictions are to be expected due to the non-resolved effects of other environmental factors and only three independent repetitions per year (three rings per CO_2_ treatment).

In addition, the most probable point estimate, i.e., the median difference, and the 50% most probable point estimates (50% posterior highest density interval (HDI)) was calculated. Basic model quality checks including convergence and effective sample sizes were performed [48,49,50], with further details given by Wohlfahrt et al. [21].

Principal component analysis (PCA) was performed using SigmaPlot version 13.0 (Systat Software Inc., San Jose, CA, USA). Within the PCA analysis, data of single FACE rings were visualized in scores plots, while the analyzed compounds were represented in corresponding loadings plots. Auto scaling was applied before calculating the model.

## 3. Results and Discussion

### 3.1. Effect of eCO_2_ on Total Soluble Solids, FT-MIR Analysis, Organic Acids and Monosccharides in Grape Must

For determination of possible differences between CO_2_ treatments, musts of the two cultivars Cabernet Sauvignon and Riesling were analyzed for various parameters with mean concentrations shown in Table 2.

Overall, it was observed that in all years and for both cultivars the ratio between tartaric and malic acid, the two most abundant organic acids, was in favour of tartaric acid within musts. Apart from 2016 when Cabernet Sauvignon tartaric acid/malic acid ratio was <1 with 0.99 for aCO_2_ and 0.95 for eCO_2_ treatment, revealing a higher malic acid than tartaric acid concentration. It was already shown in very early studies that the ratio of tartaric/malic acid (1.34 to 3.74) can be quite variable from one vintage to another [51] or even drop below 1, but it also depends on other factors like grape cultivar [52,53,54]. In the previous study, cultivars showed also different responses regarding these main acids in 2015. Whereas Cabernet Sauvignon had the highest concentration of tartaric acid in 2015, malic acid was lowest. In comparison, Riesling revealed for both organic acids the highest values in this year. Possible reasons besides the cultivar, could be the bunch structure and berry size classes which were reported to have an influence on malic acid degradation [21]. The two main monosaccharides, glucose and fructose which occur in relation 1:1 in musts of healthy grapes [55], showed ratios of 0.99 to 1.03 for Cabernet Sauvignon and 0.96 to 0.99 for Riesling over the three years. When grapes are affected by *Botrytis cinerea* the glucose to fructose ratio changes in direction of fructose, what can be assumed for Riesling must when ratios were <1, even so a selected harvest was conducted.

**Figure 2:**
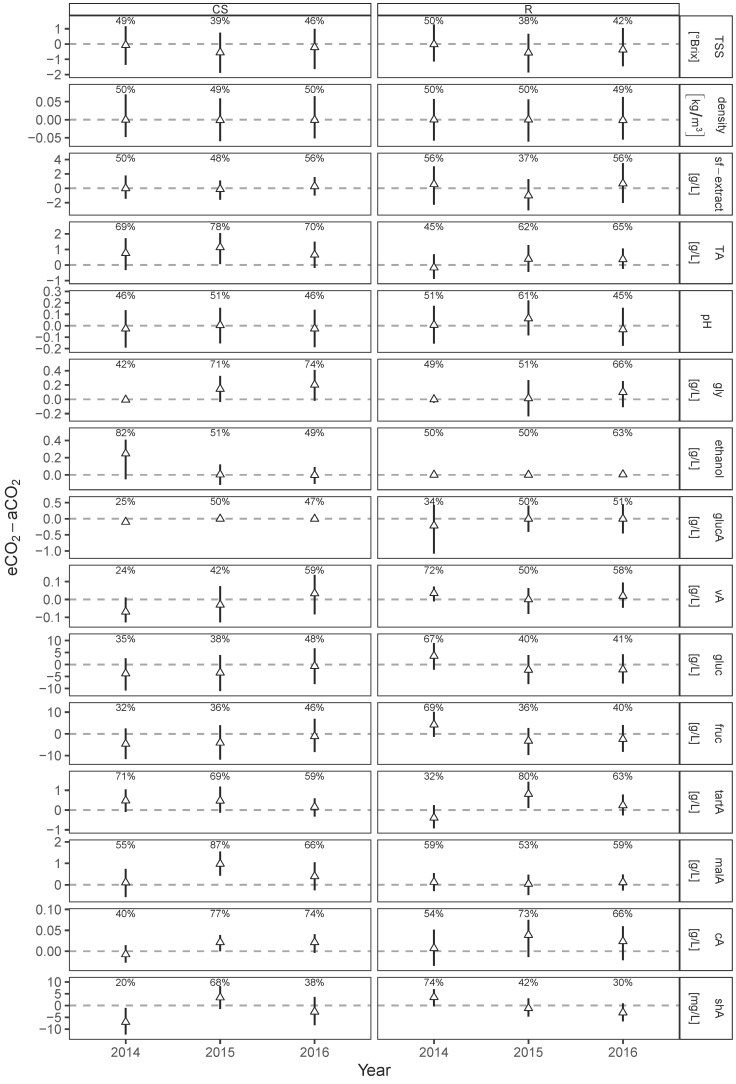
Posterior predicted difference (median and 50% HDI) between eCO_2_ and aCO_2_ for each measurement year from Bayesian generalized linear mixed effects models on analyzed must parameters of Cabernet Sauvignon (CS) and Riesling (R). Percentages represent the probability of eCO_2_-aCO_2_ > 0. Filled symbols indicate “significant differences”, if the probability is >90% (positive difference) or <10% (negative difference).

The Bayesian generalized linear mixed model analyses for must parameters was attested to not be affected by elevated CO_2_ conditions when comparing posterior predictions of the differences between eCO_2_ and aCO_2_ for both cultivars (Figure 2). No significance, i.e., 90% of the one-sided posterior predicted differences being larger (lower) than zero, were found in treatment for the three years and both cultivars. Acids and sugars in grape must at harvest were not or very little affected by eCO_2_. Similar results were obtained for cv. Sangiovese [17,18] and cv. Touriga Franca [12] under eCO_2_, even though mentioned parameters are well known to be sensitive to changing climate conditions [4,9,56]. Only in one year a higher total acidity (+12%) was detected for cv. Touriga Franca under eCO_2_ treatment [12]. For Cabernet Sauvignon, malic acid (malA) was most affected in 2015 with a probability of 87% (Figure 2) being higher under eCO_2_ conditions, even though it was not attested as “significantly different”. Nevertheless, eCO_2_ was previously accounted to increase berry weight of Cabernet Sauvignon with resulting higher malic acid concentration and increasing grape maturity [21]. The present results support these earlier findings. Within Riesling the highest positive difference between aCO_2_ and eCO_2_ was also found in 2015 but for tartaric acid with a probability of 80%. Interestingly, earlier results showed that tartaric acid in Riesling of the same vintage was found to be significant lower at beginning of ripening, but this effect disappeared with increasing maturity [21]. Present results are indicating, that this effect might even increase until harvest date. Lowest negative difference with 20% was found for shikimic acid in Riesling for the vintage 2014, but was not corroborated for the following two vintages.

However, the vintage effect was shown to have a much larger influence on must composition for both cultivars (Figure S1) than CO_2_ treatment. Cabernet Sauvignon differed slightly in vintage response from Riesling with effects on sugar free extract, total acidity, glycerol, gluconic acid, tartaric acid, malic acid, and shikimic acid. Sugar-free extract and glycerol differed between 2014 and the two other vintages and within both treatments. This was indicated by higher values in sugar-free extract and lower glycerol content in must of 2014 vintage compared to 2015 and 2016 (Table 2). The vintage 2016 was separated for total acidity, which was lower compared to 2014 and 2015. All vintages varied in gluconic and tartaric acid, thus showing the strongest vintage-dependent response on these acids. As shown in Table 2, gluconic acid was higher in 2014, whereas tartaric acid was higher in 2016. Volatile acidity differed for 2014 within eCO_2_ treatment, while 2015 vintage differed for malic and shikimic acid within aCO_2_ treatment.

Sugar-free extract of Riesling differed between 2015 and 2016 within both treatments and was higher in 2016 compared to 2015 (Table 2). Since 2015 was very dry with precipitation during vegetation period lower than 227 mm this may explain why the water soluble compounds such as sugar-free extract was the lowest. Total acidity showed lowest values in 2016 and highest amount in 2015, and were therefore separated between the two vintages. Glycerol and volatile acid differed between 2014 and the two other vintages and within both treatments, showing lower levels for both parameters in 2014. Tartaric and malic acid differed between 2015 and 2016 for both treatments with higher concentrations for both acids in 2015 vintage. Musts of Riesling were also separated in ethanol content in 2016, and within both CO_2_ treatments, showing lower levels in the respective vintage. The vintage effects were supported by climatic data of the three vegetation periods (1 April to 31 October), with lowest precipitation in 2015 (Figure 1) and higher average temperature in 2014 (+0.4 °C) compared to 2015 and 2016. Nevertheless, highest average temperatures detected in July and August 2015 were 21.9 °C and 21.5 °C, respectively. These vintage effects were reported earlier, e.g., in vegetative growth or physiological response of grapevines under CO_2_ enrichment [19].

### 3.2. Principal Component Analysis (PCA) on Must Parameters of Two Different CO_2_ Regimes

**Figure 3:**
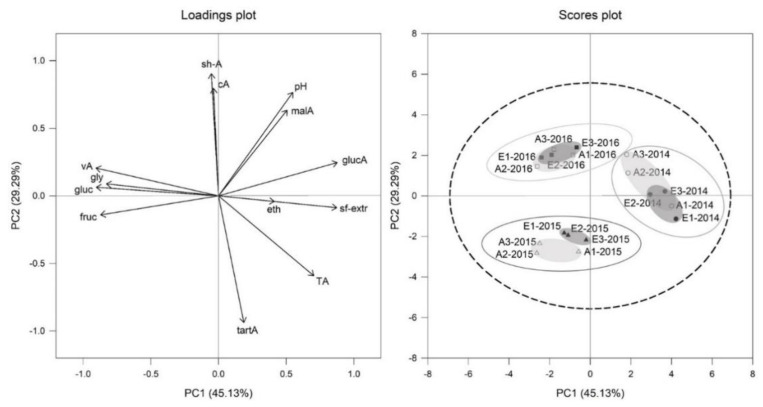
Loadings and scores plot of principal component analysis (PCA) of Cabernet Sauvignon must parameters from aCO_2_ (open symbols) and eCO_2_ (filled symbols) treatment for the years 2014 (circles), 2015 (triangles) and 2016 (squares). Data represent mean values per ring and *p* = 0.95 for confidence level (dashed line).

**Figure 4:**
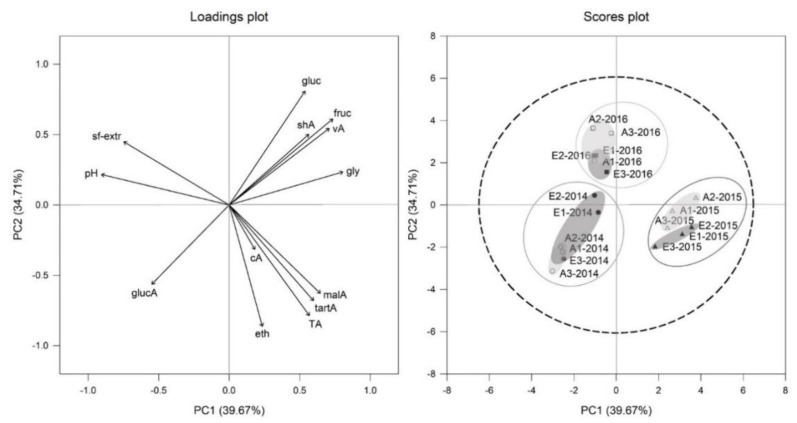
Loadings and scores plot of principal component analysis (PCA) of Riesling must parameters from aCO_2_ (open symbols) and eCO_2_ (filled symbols) treatment for the years 2014 (circles), 2015 (triangles) and 2016 (squares). Data represent values per ring and *p* = 0.95 for confidence level (dashed line).

Principal component analyses (PCA) of all must parameters for Cabernet Sauvignon (Figure 3) and Riesling (Figure 4) affirmed, that the vintage effect was larger than CO_2_ effect. This is clearly displayed by division of the three vintages 2014, 2015 and 2016 for Riesling in the scores plot of Figure 4 and for Cabernet Sauvignon in scores plot of Figure 3. It was also noticeable for both cultivars within the 2015 vintage group that treatments tended to be more separated between aCO_2_ (light grey circular area, scores plot Figure 3 and Figure 4) and eCO_2_ (dark grey circular area, scores plot Figure 3 and Figure 4) than for the other vintages.

The PC1 for Cabernet Sauvignon explained 45% and PC2 29% of the variation, characterized by elevated acid concentrations and sugar free extract on the right side, while sugars (fructose and glucose) were higher on the left side (Figure 3). Cabernet Sauvignon musts of 2014 vintage shifted to the right side and were separated by PC1 from 2015 and 2016 vintage, which were located on the left side. This could be explained by higher values for sugar-free extract and higher amount of gluconic acid in 2014 and higher glycerol concentration and higher fructose and glucose content in 2015 and 2016 musts.

The PC1 of Riesling explained 40% of the variation and was specified by elevated glycerol, sugar and acid concentrations on the right side and higher sugar free extract, pH and gluconic acid on the left side (Figure 4). Vintages of Riesling were separated by PC1, showing 2014 and 2016 shifting to the left side, while 2015 is located on the right side. 2016 vintage was additionally separated by PC2 from 2014 and 2015 vintage by shifting to the top of the plot. The differences could be explained by higher acid concentrations and glycerol in 2015 musts compared to the two other vintages which showed higher sugar-free extract and pH (Table 2).

### 3.3. Effects of eCO_2_ on FT-MIR and NMR Analysis, Total Phenolics and TEAC in Young Wines

Wines of the two cultivars and the two CO_2_ treatments were analyzed for various parameters with mean concentrations shown in Table 3.

**Figure 5:**
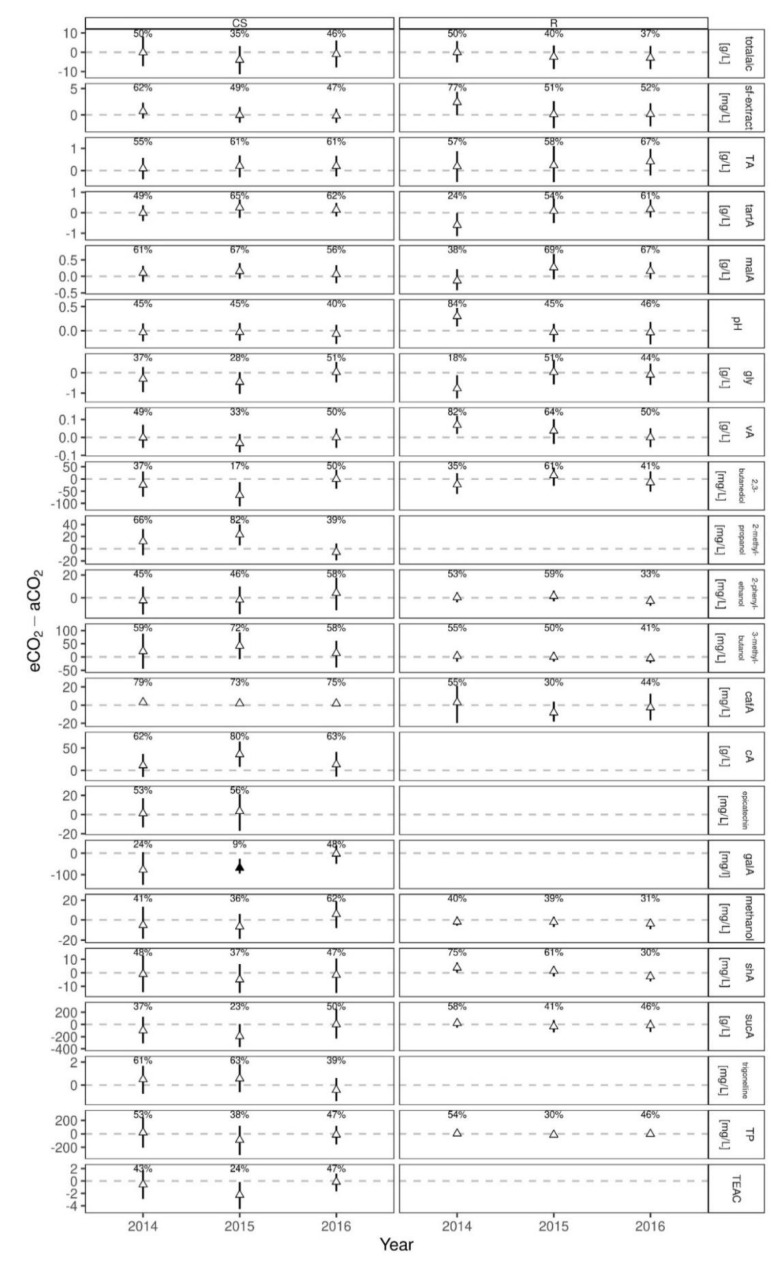
Posterior predicted difference (median and 50% HDI) between eCO_2_ and aCO_2_ for each year of measurement from Bayesian generalized linear mixed effects models on wine analyses of Cabernet Sauvignon (CS) and Riesling (R). Percentages represent the probability of eCO_2_-aCO_2_ > 0. Filled symbols indicate “significant differences”, if the probability is >90% (positive difference) or <10% (negative difference).

The Bayesian generalized linear mixed model analyses for compounds in wines was attested to be almost non-affected by eCO_2_. Posterior predictions of the differences between eCO_2_ and aCO_2_ for both cultivars are presented in Figure 5. One significance, being below 10% of the one-sided posterior predicted difference was found for Cabernet Sauvignon in 2015 for the parameter galacturonic acid, showing a lower concentration in the eCO_2_ treatment. As galacturonic acid predominantly originates through degradation of grape skin pectins and increases with grape skin contact, concentrations in German red wines ranged between 390 to 1140 mg/L [57]. This was in agreement with galacturonic acid amounts in Cabernet Sauvignon for 2014 with values between 410 to 537 mg/L, independent of CO_2_ treatment (Table 3). Concentration for vintages 2015 and 2016 seemed to be below concentration levels (166 to 269 mg/L) reported by Sponholz and Dittrich [57], but wider ranges of galacturonic acid were found in red wines of New York State (7 to 2274 mg/L) [58]. Also, galacturonic acid amount in wine was described to be influenced by other factors, e.g., year, cultivar or the use of pectic enzymes [59]. Additionally, in 2015 citric acid and 2-methyl-propanol were also affected for Cabernet Sauvignon with probabilities of 80% and 82%, respectively (Figure 5) being higher under eCO_2_ conditions, even though these were not attested as “significantly different”. The probability of citric acid in wine was in accordance to the response in must of 2015 (77%), even though the concentration marginally increased with fermentation. An increase of citric acid during fermentation was reported earlier and can potentially double [60]. When comparing previous results of young red Italian and Portuguese wines [12,17,18], total acidity, volatile acidity, 2-phenylethanol and 3-methyl-butanol were not affected by eCO_2_ level, which was confirmed in the present study (Figure 5). No differences between aCO_2_ and eCO_2_ treatment in pH and total phenolics were at least confirmed for wines of cv. Sangiovese [17,18] and in one season for cv. Touriga Franca [12]. In contrast, an inhibition of total phenolics was detected for cv. Touriga Franca wines of another season [12], which was also noticeable in the warmer vintage 2015, when TEAC and total phenolics of Cabernet Sauvignon showed lower levels under eCO_2_ when compared to 2015 and 2016, even though without a significant difference.

For Riesling the highest positive differences between aCO_2_ and eCO_2_ were found in 2014 for pH and volatile acidity with probabilities of 84% and 82%, respectively (Figure 5). Also in 2014, the lowest negative difference with 18% was found for glycerol in Riesling. These findings for highest and lowest probabilities within Riesling were not consistent for the following years and are in accordance with emerging vintage effects.

Also in young wines, vintage was shown to have a larger effect on the composition for both cultivars (Figure S2) than CO_2_ treatment. Not all vintage responses found in must were transferred into the young wines. Vintage responses of Cabernet Sauvignon showed effects on numerous chemical parameters like total alcohol, sugar free extract, tartaric acid, glycerol, volatile acid, 2,3-butanediol, 2-methyl-propanol, 3-methyl-butanol, caftaric acid, galacturonic acid, shikimic acid, succinic acid, trigonelline, total phenolics and TEAC. Total alcohol content differed between 2014 and 2016 within eCO_2_ treatment and nearly within aCO_2_ (89%), with higher total alcohol content in 2014 and lower total alcohol in 2016 as shown in Table 3. 2016 was separated from the two other vintages and for both treatments in sugar free extract by showing lower concentrations. Interestingly, in must it was 2014 vintage that appeared with higher values in sugar-free extract. For tartaric acid, 2016 differed within aCO_2_ treatment, whereas within eCO_2_ 2015 was different. Vintage responses for total acidity and malic acid found in must disappeared in young wines. Glycerol in wines, formed during alcoholic fermentation was separated for 2014 with higher values in that year. Volatile acidity differed between 2014 and 2015 within eCO_2_ treatment, for the same treatment succinic acid differed between 2015 and 2016. The 2016 vintage was separated for 2,3-butanediol, but only within aCO_2_. 2-methyl-propanol was different in 2014 for aCO_2_ and 2016 within eCO_2_, whereas for 3-methyl-butanol in 2014 was separated within aCO_2_ and a response close to 90% was shown for eCO_2_ (Figure S2). Galacturonic acid differed in 2014 within aCO_2_ and between all vintages within eCO_2_. This is in accordance with the above mentioned significant difference between CO_2_ treatments. Vintage response for shikimic acid showed a separation of 2015 for eCO_2_ with a lower concentration and a close response of 12% within the aCO_2_ treatment. All vintages varied in caftaric acid, showing the strongest vintage-dependent response within Cabernet Sauvignon young wine. Trigonelline, total phenolics and TEAC were separated for 2016 with lower values compared to the other vintages.

Riesling vintage responses were different from Cabernet Sauvignon especially regarding acidity parameters. For example, total acidity differed between all vintages and for both treatments showing higher values in 2015 and lower values in 2016. Tartaric and malic acid were both separated for 2016 vintage and within the two treatments, when concentrations were lower compared to 2014/15. Volatile acidity differed between 2015 and the two other vintages, showing higher values in 2015, whereas succinic acid was separated for 2014 with lower concentrations for both treatments. The pH differed between 2016 and 2014/15 for aCO_2_ and between 2015 and 2016 for eCO_2_, revealing pH of ≥3.0 in 2016. For caftaric acid only for eCO_2_ a difference was detected between 2014 and 2015. Total phenolics were close to be separated for both treatments and 2014 vintage (Figure S2).

### 3.4. Effects of eCO_2_ on Riesling and Cabernet Sauvignon Colorimetric Parameters in Young Wines

Results of the colorimetric analyses for both cultivars are presented in Table 4, anthocyanins and monomeric indices of the red cultivar Cabernet Sauvignon are shown in Table 5.

**Figure 6:**
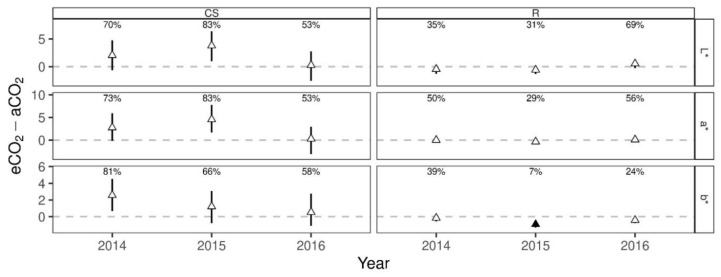
Posterior predicted difference (median and 50% HDI) between eCO_2_ and aCO_2_ for each measurement year from Bayesian generalized linear mixed effects models on CIELab parameters of Cabernet Sauvignon (CS) and Riesling (R). Percentages represent the probability of eCO_2_-aCO_2_ > 0. Filled symbols indicate “significant differences”, if the probability is >90% (positive difference) or <10% (negative difference).

Elevated CO_2_ had no effect on color parameters L* (lightness), a* (green-red) and b* (blue-yellow) of Cabernet Sauvignon, even though values were slightly higher in 2014 and 2015 (Figure 6). This was also shown by Gonçalves et al. [12] when analyzing berry color of cv. Touriga Franca, which did not result in significant changes under eCO_2_, except in one out of two years, when b* was significantly higher. As higher b* leads to less blueness in color [61], this corresponded with significantly decreased anthocyanin content under eCO_2_ in the later wines of Touriga Franca in that year [12]. Independent of treatment, similar results were shown for Cabernet Sauvignon between 2014 and the two other vintages, when lower L* and a* were related to higher total anthocyanin content in wines.

During aging of red wines there is a remarkable change in color, mainly due to a decrease in L*, and by showing a darker color [62]. In relation to L* values found in aCO_2_ and eCO_2_ of Cabernet Sauvignon young wines, a tendency to lower values under aCO_2_ (Table 4) was apparent. Therefore, a darker color could be assumed and needs to be further investigated in respect to the aging potential of wines from these two treatments. But various other factors need to be taken in account when conducting wine aging trials, e.g., temperature, pH or acetaldehyde concentration in wines [63,64,65].

In comparison, Riesling showed a significant difference for b* in 2015, being below 10% of the one-sided posterior predicted difference (Figure 6) with lower b* under eCO_2_. In white wines, b* values were reported by Blesic et al. to decrease with filtration, showing means of 6.93 for unfiltered and 4.00 for filtered white wines [66]. Wines in the present study were analyzed prior filtration, but after clarification, b* ranged from 4.49 to 6.40 representing a similar range. Also, high values of L* were detected for both treatments and all vintages (Table 4), showing close to 100% of quantity of transmitted global light and can therefore be quantified as a pale yellow color [67], typically for young Riesling wines. As during storage of white wines an alteration of a* and b* is described, resulting in a change of color from pale yellow to yellow-brown [67] it would be of further interest to follow up the colorimetric development of the two CO_2_ treatments for Riesling.

Colorimetric parameters differed between vintages as well and for both cultivars (Figure S3). Cabernet Sauvignon differed between all vintages in L* and a* within aCO_2_, showing lower values in 2014 and higher values in 2016. Also within aCO_2_, b* was different in 2015. For the eCO_2_ treatment 2014 vintage was different from 2015/2016 for L* and a*, whereas b* differed between 2015 and 2016. Vintage responses of L*, a* and b* were already reported by Gonçalves et al. [12] for the red cultivar Touriga Franca under different CO_2_ treatments.

For colorimetric parameters Riesling was less sensitive to vintage effects. L* differed between 2014 and 2015/2016 within aCO_2_ and between 2014 and 2015 for eCO_2_ with higher values for both treatments in 2014. The same response was found for b* with the distinction that lower values were detected in 2014 and both treatments.

### 3.5. Anthocyanin and Monomeric Index Response of Cabernet Sauvignon Young Wines to eCO_2_ Treatment

**Figure 7:**
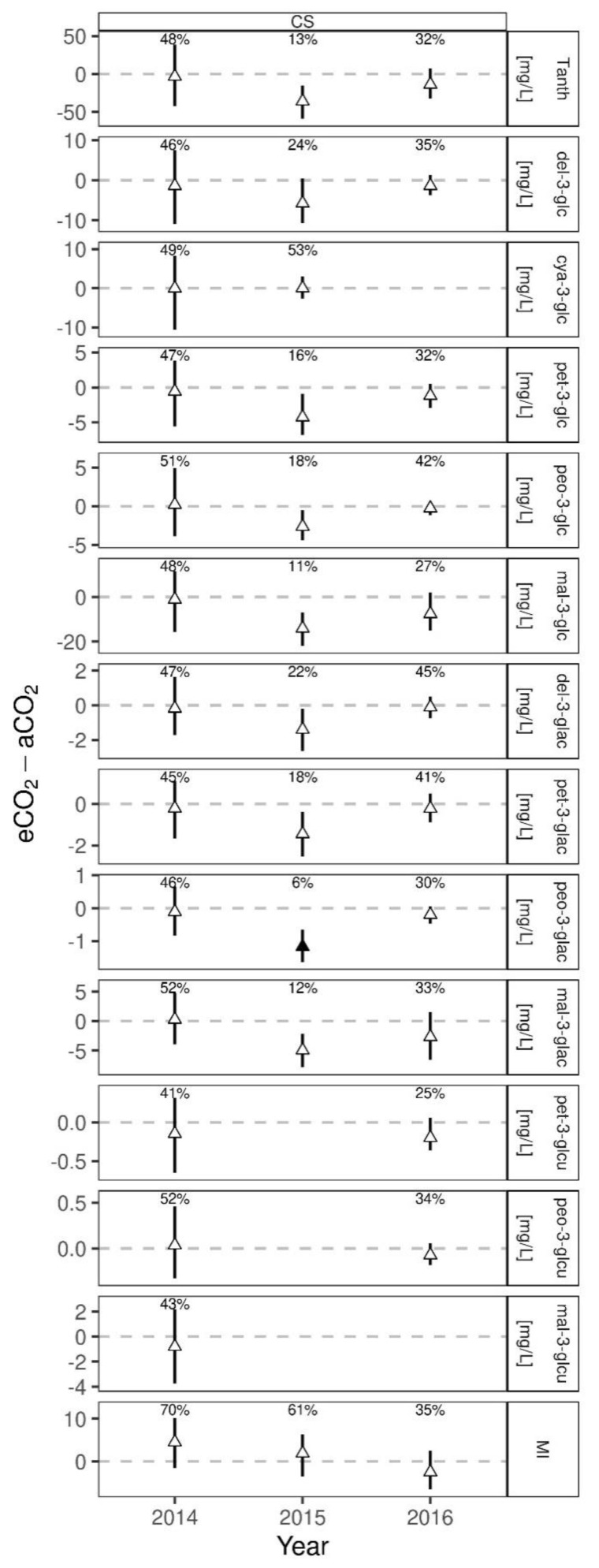
Posterior predicted difference (median and 50% HDI) between eCO_2_ and aCO_2_ for each measurement year from Bayesian generalized linear mixed effects models on anthocyanins and monomeric index (MI) in Cabernet Sauvignon (CS) wines. Percentages represent the probability of eCO_2_-aCO_2_ > 0. Filled symbols indicate “significant differences”, if the probability is >90% (positive difference) or <10% (negative difference).

Results of the HPLC analysis of anthocyanins and monomeric indices of Cabernet Sauvignon young wines for the three vintages and two treatments are presented in Table 5. In 2014, the first year of CO_2_ enrichment within the VineyardFACE, the one-sided posterior predicted difference was 48%, meaning no difference between the two treatments in total anthocyanin concentration. Interestingly, all analyzed anthocyanins were more or less not affected in 2014, confirmed with the posterior predictive differences ranging between 41% for petunidin-3-*O*-(6”-p-coumaroyl)-glucoside to 52% for malvidin-3-*O*-(6”-acetyl)-glucoside and peonidin-3-*O*-(6”-p-coumaroyl)-glucoside (Figure 7). A similar response was already reported for single berry weight during berry development and yield for the year 2014 and was closely linked to inflorescence initiation, which took place when CO_2_ enrichment was not initiated yet [19,21]. Compared to 2015 and 2016 with predicted differences of 13% and 32% respectively, a tendency to a lower total anthocyanin concentration under eCO_2_ can be assumed for the following years. In wines of Touriga Franca a significantly decrease of total anthocyanins was detected under eCO_2_ [12]. Associated with the increased berry weights of Cabernet Sauvignon found in 2015 and 2016 under eCO_2_ [21], higher berry weights are described to result in lower concentrations of grape skin located compounds like anthocyanins [22] and could therefore emerge as an indirect CO_2_ effect.

Malvidin-3-*O*-glucoside, delphinidin-3-*O*-glucoside, petunidin-3-*O*-glucoside, peonidin-3-*O*-glucoside, and cyanidin-3-*O*-glucoside in the listed order are the main monomeric anthocyanins, including their derivatives, which are found in red wines of *Vitis vinifera* [68,69,70,71]. This was confirmed for all vintages of Cabernet Sauvignon. Most abundant in Cabernet Sauvignon was malvidin-3-*O*-glucoside and its derivatives showing higher concentrations compared to other anthocyanins (Table 5). Lowest values were found for cyanidin-3-*O*-glucoside in 2014 and 2015, and non-detectable values in 2016 for both CO_2_ treatments. The acylated and p-coumaroylated form of cyanidin-3-*O*-glucoside was not detected. As cyanidin-3-*O*-glucoside is highly reactive and known for its low initial amount in grape extracts, its concentration is marginal in red wines and also in Cabernet Sauvignon [71,72,73]. The acylated form was found for malvidin-3-*O*-glucoside, delphinidin-3-*O*-glucoside, petunidin-3-*O*-glucoside and peonidin-3-*O*-glucoside, but the p-coumaroylated form was only found for malvidin-3-*O*-glucoside, peonidin-3-*O*-glucoside, and petunidin-3-*O*-glucoside. In Bordeaux cultivars like Cabernet Sauvignon these two forms were described to occur in a ratio of 2:1 [74], which was similar in our study. A significant difference for peonidin-3-*O*-(6”-acetyl)-glucoside was found in 2015, with a one-sided posterior predicted difference of 6% (Figure 7) being lower under eCO_2_ treatment. Also close to a significant difference with 11% and 12% was malvidin-3-*O*-glucoside and malvidin-3-*O*-(6”-acetyl)-glucoside. These lower levels of anthocyanins under eCO_2_ appeared in the warmest season 2015 and needs to be further investigated for following vintages with higher daily temperatures, as anthocyanin accumulation is known to be highly dependent on temperature and light intensity [2,75,76,77,78]. Total phenolics of red wines in 2015 tended to similar results and point towards a possible future change in phenolic and anthocyanin concentration under a combination of warm seasons and eCO_2_ in a temperate oceanic climate.

### 3.6. Principal Component Analysis (PCA) on Young Wines of Two Different CO_2_ Regimes

**Figure 8:**
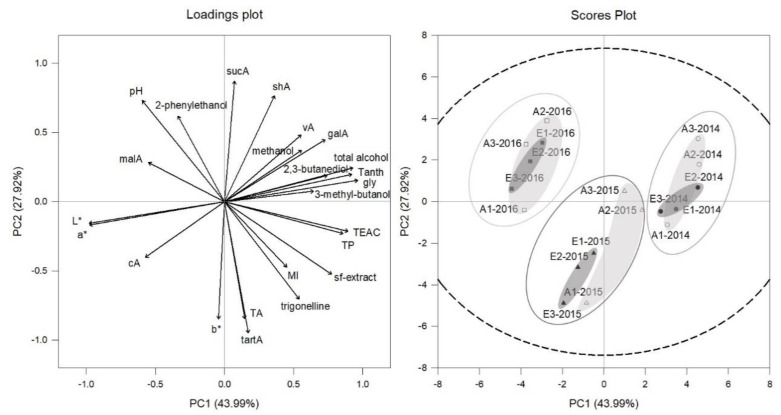
Loadings and scores plot of principal component analysis (PCA) of Cabernet Sauvignon young wines from aCO_2_ (open symbols) and eCO_2_ (filled symbols) treatment for the years 2014 (circles), 2015 (triangles) and 2016 (squares). Data represent mean values per ring and *p* = 0.95 for confidence level (dashed line).

**Figure 9:**
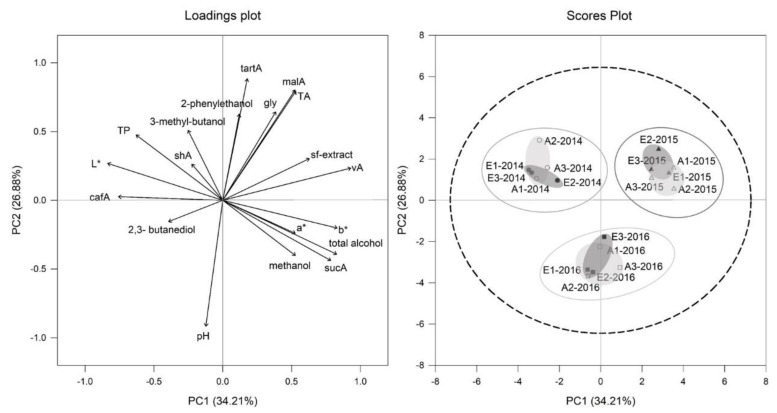
Loadings and scores plot of principal component analysis (PCA) of Riesling young wines from aCO_2_ (open symbols) and eCO_2_ (filled symbols) treatment for the years 2014 (circles), 2015 (triangles) and 2016 (squares). Data represent mean values per ring and *p* = 0.95 for confidence level (dashed line).

Principal component analysis (PCA) of selected wine parameters for Cabernet Sauvignon (Figure 8) and Riesling (Figure 9) confirmed that the vintage effect found in must is larger than the CO_2_ effect. In the scores plots of Figure 8 and Figure 9 it is displayed how vintages 2014, 2015 and 2016 for Cabernet Sauvignon and Riesling are separated. Again, for both cultivars within 2015, vintage treatments tended to be more differentiated between aCO_2_ (light grey circular area, scores plot Figure 8 and Figure 9) and eCO_2_ (dark grey circular area, scores plot Figure 8 and Figure 9) than for the other vintages.

The PC1 for Cabernet Sauvignon explained 44% and PC2 28% of the variation, characterized by elevated total and higher alcohols, glycerol, total phenolics and anthocyanins, galacturonic acid and sugar free extract on the right side. However, malic and citric acid, L*, a* and pH were higher on the left side (Figure 8). Cabernet Sauvignon young wines of 2014 vintage shifted to the right side and were therefore separated by PC1 from 2016 vintage located on the left side. This was explained amongst others by higher values of total alcohol, glycerol and total anthocyanins in 2014 and higher malic acid, L* and a* in 2016 wines. This linkage between CIELab parameters and anthocyanins was already reported for Cabernet Sauvignon young wines by Han et al. [79].

The PC1 of Riesling explained 34% of the variation and was specified by elevated higher acid concentrations, sugar free extract, higher total alcohol and a* and b* on the right side and higher total phenolics, caftaric acid and L* on the left side (Figure 9). Riesling vintages of 2014 and 2015 were separated by PC1, showing 2014 on the left side, while 2015 was located on the right side. The 2016 vintage was separated by PC2 from 2014 and 2015 vintage by shifting to the bottom of the plot. Differences could be explained by higher malic acid concentration, sugar free extract and volatile acidity in 2015 compared to the two other vintages which were higher in total phenolics and L* (2014) or pH (2016) (Table 3).

## 4. Conclusions

In summary, the present study provides evidence that a predicted increase in future CO_2_ concentration (+20% of the current atmospheric CO_2_ concentration), which is known to highly impact on grapevines physiology and vegetative as well as generative performance [19,20,21], has only a little effect on grape must and young wine composition under a temperate ocean climate. Furthermore, for the first time a white cultivar was investigated in a free air enrichment system regarding the compositional quality of must and wine and was proofed to be not negatively affected by an eCO_2_ concentration. In fact, the results presented can give a first evidence to a long-term study but needs to be continued in investigation of the aging behavior of existing wines and continuous analysis of wines produced from upcoming vintages.

## Figures and Tables

**Table 1:** Investigated compounds subdivided by analytical methods.

Analyzed Compound	Abbreviation	Analytical Method	Medium
total soluble solids	TSS	refractometry	must
density 20/20		FT-MIR	must
sugar-free extract	sf-extr		must, wine
total acidity	TA		must, wine
tartaric acid	tartA		wine
malic acid	malA		wine
pH			must, wine
glycerol	gly		must, wine
ethanol			must
gluconic acid	glucA		must
volatile acid	vA		must, wine
actual alcohol	alc		wine
residual sugar	sugar		wine
glucose	gluc	HPLC	must
fructose	fruc		must
tartaric acid	tartA		must
malic acid	malA		must
citric acid	cA		must
shikimic acid	shA		must
2,3-butanediol		NMR	wine
2-methyl-propanol			wine ^1^
2-phenylethanol			wine
3-methyl-butanol			wine
caftaric acid	cafA		wine
citric acid	cA		wine ^1^
epicatechin			wine ^1^
galacturonic acid	galA		wine ^1^
methanol			wine
shikimic acid	shA		wine
succinic acid	sucA		wine
trigonelline			wine ^1^
total phenolics	TP	spectrophotometry	wine
trolox equivalent antioxidative capacity	TEAC	spectrophotometry	wine ^1^
CIELab coordinates	L*, a*, b*	spectrophotometry	wine
monomeric index	MI	spectrophotometry	wine ^1^
total anthocyanins	Tanth	HPLC	wine ^1^
delphinidin-3-*O*-glucoside	del-3-glc		wine ^1^
cyanidin-3-*O*-glucoside	cya-3-glc		wine ^1^
petunidin-3-*O*-glucoside	pet-3-glc		wine ^1^
peonidin-3-*O*-glucoside	peo-3-glc		wine ^1^
malvidin-3-*O*-glucoside	mal-3-glc		wine ^1^
delphinidin-3-*O*-(6″-acetyl)-glucoside	del-3-glac		wine ^1^
petunidin-3-*O*-(6″-acetyl)-glucoside	pet-3-glac		wine ^1^
peonidin-3-*O*-(6″-acetyl)-glucoside	peo-3-glac		wine ^1^
malvidin-3-*O*-(6″-acetyl)-glucoside	mal-3-glac		wine ^1^
petunidin-3-*O*-(6″-p-coumaroyl)-glucoside	pet-3-glcu		wine ^1^
peonidin-3-*O*-(6″-p-coumaroyl)-glucoside	peo-3-glcu		wine ^1^
malvidin-3-*O*-(6″-p-coumaroyl)-glucoside	mal-3-glcu		wine ^1^

^1^ Wine analysis conducted only for red cultivar Cabernet Sauvignon.

**Table 2:** Parameters of Cabernet Sauvignon and Riesling must analysis at two CO_2_ levels (aCO_2_ and eCO_2_) at harvest date in 2014, 2015 and 2016.

	Cabernet Sauvignon						Riesling					
**year**	2014		2015		2016		2014		2015		2016	
**treatment**	aCO_2_	eCO_2_	aCO_2_	eCO_2_	aCO_2_	eCO_2_	aCO_2_	eCO_2_	aCO_2_	eCO_2_	aCO_2_	eCO_2_
TSS [°Brix]	19.1 ± 0.9	19.1 ± 0.8	20.4 ± 0.8	19.8 ± 0.5	19.8 ± 0.5	19.5 ± 0.5	19.6 ± 0.4	19.6 ± 0.5	21.0 ± 0.5	20.5 ± 0.8	20.5 ± 0.4	20.2 ± 0.0
density 20/20	1.082 ± 0.004	1.081 ± 0.004	1.089 ± 0.003	1.086 ± 0.002	1.083 ± 0.002	1.083 ± 0.002	1.082 ± 0.001	1.082 ± 0.002	1.091 ± 0.002	1.090 ± 0.002	1.087 ± 0.001	1.086 ± 0.000
sf-extract [g/L]	27.2 ± 0.5	27.2 ± 0.9	22.7 ± 0.1	22.5 ± 0.2	21.0 ± 0.7	21.3 ± 0.1	25.1 ± 2.2	25.8 ± 3.5	21.0 ± 0.9	20.0 ± 0.9	26.2 ± 1.1	26.9 ± 0.4
TA [g/L]	13.20 ± 0.56	13.98 ± 0.57	12.63 ± 0.49	13.77 ± 0.21	10.92 ± 0.83	11.57 ± 0.60	11.52 ± 0.38	11.37 ± 0.06	12.50 ± 0.53	12.87 ± 0.15	9.37 ± 0.55	9.73 ± 0.42
pH	3.00 ± 0.02	2.98 ± 0.03	2.77 ± 0.06	2.77 ± 0.06	2.95 ± 0.02	2.93 ± 0.02	2.93 ± 0.02	2.94 ± 0.01	2.67 ± 0.06	2.73 ± 0.06	2.93 ± 0.06	2.90 ± 0.00
gly [g/L]	0.13 ± 0.01	0.12 ± 0.00	0.97 ± 0.23	1.10 ± 0.10	1.16 ± 0.19	1.35 ± 0.22	0.12 ± 0.01	0.12 ± 0.01	0.67 ± 0.15	0.67 ± 0.12	0.44 ± 0.22	0.52 ± 0.10
ethanol [g/L]	0.12 ± 0.00	0.28 ± 0.27	0.10 ± 0.00	0.10 ± 0.00	0.08 ± 0.01	0.08 ± 0.00	0.10 ± 0.01	0.10 ± 0.00	0.10 ± 0.00	0.10 ± 0.00	0.07 ± 0.00	0.07 ± 0.01
glucA [g/L]	0.53 ± 0.01	0.45 ± 0.13	0.10 ± 0.00	0.10 ± 0.00	0.16 ± 0.01	0.16 ± 0.01	0.57 ± 0.02	0.19 ± 0.33	0.17 ± 0.06	0.13 ± 0.06	0.16 ± 0.01	0.16 ± 0.01
vA [g/L]	0.30 ± 0.07	0.23 ± 0.07	0.40 ± 0.10	0.37 ± 0.06	0.41 ± 0.05	0.44 ± 0.05	0.23 ± 0.06	0.26 ± 0.02	0.43 ± 0.06	0.43 ± 0.06	0.41 ± 0.04	0.43 ± 0.01
gluc [g/L]	91.1 ± 6.8	87.5 ± 4.2	99.0 ± 4.6	95.6 ± 3.7	97.8 ± 2.7	97.3 ± 3.5	91.4 ± 1.2	95.0 ± 3.5	100.2 ± 2.3	97.9 ± 2.6	101.9 ± 1.8	99.9 ± 0.2
fruc [g/L]	90.1 ± 7.7	85.3 ± 3.7	100.5 ± 5.4	96.2 ± 3.6	95.8 ± 4.4	94.7 ± 3.8	93.1 ± 1.1	97.6 ± 3.6	104.7 ± 3.0	101.3 ± 3.2	102.9 ± 1.8	100.6 ± 0.3
tartA [g/L]	7.26 ± 0.79	7.74 ± 0.21	8.55 ± 0.52	9.03 ± 0.20	5.95 ± 0.40	6.11 ± 0.13	7.78 ± 0.20	7.39 ± 0.18	8.24 ± 0.29	9.05 ± 0.55	6.73 ± 0.48	6.97 ± 0.08
malA [g/L]	6.30 ± 0.30	6.43 ± 0.24	4.85 ± 0.32	5.82 ± 0.25	6.04 ± 0.87	6.45 ± 0.80	3.91 ± 0.21	4.06 ± 0.26	4.47 ± 0.22	4.51 ± 0.21	3.49 ± 0.35	3.62 ± 0.38
cA [g/L]	0.22 ± 0.02	0.22 ± 0.01	0.20 ± 0.01	0.22 ± 0.00	0.23 ± 0.02	0.25 ± 0.01	0.17 ± 0.00	0.17 ± 0.01	0.16 ± 0.01	0.19 ± 0.05	0.15 ± 0.03	0.17 ± 0.03
shA [mg/L]	69.1 ± 7.1	61.9 ± 4.9	54.4 ± 2.2	57.8 ± 2.0	71.3 ± 3.8	68.7 ± 2.2	42.7 ± 1.8	46.3 ± 2.7	47.9 ± 1.7	46.7 ± 3.4	48.0 ± 3.3	45.0 ± 0.9

Values are the mean (±SD) of three FACE rings per treatment with aCO_2_ (A1, A2 and A3) and eCO_2_ (E1, E2 and E3).

**Table 3:** Results of cvs. Cabernet Sauvignon and Riesling wine analysis at two CO_2_ levels (aCO_2_ and eCO_2_) after fermentation of vintages 2014, 2015 and 2016.

	Cabernet Sauvignon						Riesling					
**year**	2014		2015		2016		2014		2015		2016	
**treatment**	aCO_2_	eCO_2_	aCO_2_	eCO_2_	aCO_2_	eCO_2_	aCO_2_	eCO_2_	aCO_2_	eCO_2_	aCO_2_	eCO_2_
total alcohol [g/L]	105.83 ± 5.02	106.06 ± 4.88	99.96 ± 5.94	95.87 ± 4.37	93.73 ± 2.65	92.85 ± 2.48	91.96 ± 1.55	91.99 ± 2.14	102.25 ± 3.49	99.80 ± 2.56	101.33 ± 1.58	98.52 ± 0.18
sf-extract [g/L]	24.9 ± 0.6	25.6 ± 0.4	24.7 ± 0.7	24.7 ± 0.8	21.9 ± 0.7	21.7 ± 0.5	19.0 ± 3.4	21.5 ± 0.3	23.4 ± 1.2	23.5 ± 0.4	19.9 ± 0.5	20.1 ± 0.5
TA [g/L]	6.93 ± 0.25	7.03 ± 0.13	7.13 ± 0.47	7.33 ± 0.15	6.67 ± 0.29	6.87 ± 0.12	10.90 ± 0.41	11.08 ± 0.10	12.66 ± 0.35	12.88 ± 0.11	9.17 ± 0.38	9.57 ± 0.21
tartA [g/L]	3.20 ± 0.48	3.22 ± 0.10	3.67 ± 0.50	3.90 ± 0.10	2.73 ± 0.40	2.87 ± 0.15	5.94 ± 0.92	5.28 ± 0.07	5.92 ± 0.37	5.94 ± 0.08	4.27 ± 0.35	4.43 ± 0.06
malA [g/L]	2.34 ± 0.25	2.43 ± 0.05	2.27 ± 0.06	2.43 ± 0.32	2.63 ± 0.15	2.70 ± 0.20	3.72 ± 0.21	3.62 ± 0.14	4.33 ± 0.12	4.57 ± 0.10	2.87 ± 0.25	3.03 ± 0.32
pH	3.25 ± 0.11	3.24 ± 0.03	3.23 ± 0.06	3.20 ± 0.00	3.47 ± 0.06	3.40 ± 0.00	2.41 ± 0.27	2.65 ± 0.00	2.46 ± 0.07	2.46 ± 0.05	3.03 ± 0.06	3.00 ± 0.00
gly [g/L]	8.80 ± 0.37	8.53 ± 0.17	7.83 ± 0.47	7.37 ± 0.25	7.07 ± 0.21	7.10 ± 0.17	6.09 ± 0.72	5.33 ± 0.17	6.09 ± 0.33	6.14 ± 0.27	5.40 ± 0.30	5.30 ± 0.26
vA [g/L]	0.38 ± 0.01	0.37 ± 0.01	0.33 ± 0.06	0.30 ± 0.00	0.33 ± 0.06	0.33 ± 0.06	0.44 ± 0.01	0.46 ± 0.01	0.62 ± 0.02	0.66 ± 0.02	0.50 ± 0.00	0.50 ± 0.00
2,3-butanediol [mg/L]	294.50 ± 37.03	269.67 ± 23.22	303.44 ± 45.35	233.75 ± 30.06	219.20 ± 52.45	217.84 ± 23.32	290.42 ± 31.70	267.98 ± 39.77	240.70 ± 37.71	257.47 ± 55.66	281.43 ± 3.14	267.91 ± 6.27
2-methyl-propanol [mg/L]	149.17 ± 9.83	162.17 ± 17.21	110.26 ± 15.30	133.15 ± 6.08	103.77 ± 1.95	94.79 ± 18.07	-	-	-	-	-	-
2-phenylethanol [mg/L]	71.67 ± 3.75	69.50 ± 4.09	71.28 ± 12.94	69.74 ± 16.86	79.53 ± 8.14	84.02 ± 1.72	39.00 ± 1.67	39.41 ± 2.08	38.87 ± 2.05	40.40 ± 3.31	37.74 ± 3.10	35.01 ± 4.89
3-methyl-butanol [mg/L]	474.67 ± 12.11	498.50 ± 21.47	354.49 ± 38.83	394.54 ± 38.42	372.53 ± 57.90	383.49 ± 44.14	154.37 ± 6.39	157.22 ± 2.49	145.26 ± 21.96	143.69 ± 4.32	141.78 ± 12.75	135.55 ± 21.03
cafA [mg/L]	27.83 ± 1.61	31.17 ± 1.89	23.83 ± 0.52	25.71 ± 1.26	17.48 ± 0.99	19.11 ± 0.71	61.58 ± 3.92	65.96 ± 9.35	39.83 ± 18.58	29.97 ± 10.33	48.45 ± 13.31	44.67 ± 3.61
cA [g/L]	0.30 ± 0.02	0.32 ± 0.02	0.32 ± 0.02	0.35 ± 0.1	0.33 ± 0.04	0.34 ± 0.04	-	-	-	-	-	-
epicatechin [mg/L]	41.7 ± 19.7	43.2 ± 17.9	46.3 ± 3.7	51.4 ± 10.4	-	-	-	-	-	-	-	-
galA [mg/L]	504.50 ± 36.34	425.33 ± 25.27	237.39 ± 37.07	166.26 ± 3.10	268.59 ± 48.92	264.97 ± 43.52	-	-	-	-	-	-
methanol [mg/L]	97.83 ± 4.54	92.83 ± 1.26	77.39 ± 5.34	70.27 ± 3.77	76.25 ± 24.48	81.15 ± 5.71	37.95 ± 3.60	36.12 ± 2.26	42.24 ± 2.92	40.24 ± 5.08	44.29 ± 3.20	40.49 ± 3.68
shA [mg/L]	135.83 ± 6.53	135.17 ± 5.35	111.77 ± 13.16	106.04 ± 1.25	129.43 ± 16.67	127.62 ± 8.70	44.12 ± 2.70	47.95 ± 1.52	43.96 ± 0.93	45.38 ± 1.42	44.90 ± 4.37	42.03 ± 0.88
sucA [g/L]	0.99 ± 0.19	0.88 ± 0.11	0.92 ± 0.32	0.69 ± 0.12	1.04 ± 0.23	1.04 ± 0.22	0.60 ± 0.04	0.63 ± 0.07	0.83 ± 0.05	0.80 ± 0.12	0.81 ± 0.07	0.79 ± 0.01
trigonelline [mg/L]	13.5 ± 0.9	14.0 ± 0.5	13.4 ± 1.3	13.9 ± 1.2	11.4 ± 0.5	11.1 ± 1.0	-	-	-	-	-	-
TP [mg/L]	1853.3 ± 206.4	1870.0 ± 179.0	1859.2 ± 320.9	1749.3 ± 109.0	1177.7 ± 45.3	1164.3 ± 31.1	211.3 ± 6.7	215.3 ± 9.8	180.0 ± 25.9	164.7 ± 15.8	165.0 ± 9.3	162.5 ± 4.1
TEAC	21.8 ± 1.9	21.2 ± 1.2	21.4 ± 3.5	19.0 ± 1.0	13.4 ± 0.2	13.3 ± 0.3	-	-	-	-	-	-

Values are the mean (± SD) of three FACE rings per treatment with aCO_2_ (A1, A2 and A3) and eCO_2_ (E1, E2 and E3). Total alcohol in wine was calculated by the sum of actual alcohol and potential alcohol (residual sugar).

**Table 4:** CIELab parameters of cvs. Cabernet Sauvignon and Riesling young wines at two CO_2_ levels (aCO_2_ and eCO_2_) after fermentation of 2014, 2015 and 2016.

	Cabernet Sauvignon						Riesling					
**year**	2014		2015		2016		2014		2015		2016	
**treatment**	aCO_2_	eCO_2_	aCO_2_	eCO_2_	aCO_2_	eCO_2_	aCO_2_	eCO_2_	aCO_2_	eCO_2_	aCO_2_	eCO_2_
L*	10.94 ± 2.43	12.98 ± 1.97	17.35 ± 3.93	21.21 ± 2.24	23.77 ± 2.06	24.05 ± 2.30	99.47 ± 0.18	99.00 ± 0.72	97.51 ± 0.73	96.93 ± 0.59	97.47 ± 0.57	98.00 ± 0.41
a*	41.39 ± 3.28	44.14 ± 2.30	49.31 ± 4.67	53.85 ± 2.17	56.59 ± 1.97	56.93 ± 2.05	−0.91 ± 0.10	−0.90 ± 0.05	−0.32 ± 0.73	−0.66 ± 0.12	−0.66 ± 0.04	−0.57 ± 0.10
b*	41.82 ± 3.30	44.30 ± 1.10	45.72 ± 3.39	46.90 ± 0.98	42.13 ± 2.43	42.62 ± 1.48	4.84 ± 0.32	4.69 ± 0.08	6.40 ± 0.43	5.50 ± 0.12	5.79 ± 0.41	5.38 ± 0.19

Values are the mean (± SD) of three FACE rings per treatment with aCO_2_ (A1, A2 and A3) and eCO_2_ (E1, E2 and E3).

**Table 5:** Anthocyanins and monomeric index (MI) in cvs. Cabernet Sauvignon young wines at two CO_2_ levels (aCO_2_ and eCO_2_) of vintages 2014, 2015 and 2016.

	Cabernet Sauvignon					
**year**	2014		2015		2016	
**treatment**	aCO_2_	eCO_2_	aCO_2_	eCO_2_	aCO_2_	eCO_2_
Tanth [mg/L]	403.73 ± 28.67	400.89 ± 32.88	228.62 ± 25.74	191.47 ± 4.71	200.38 ± 10.20	186.70 ± 13.10
del-3-glc [mg/L]	53.31 ± 7.84	52.13 ± 9.31	34.95 ± 8.71	28.60 ± 2.30	14.92 ± 2.13	13.54 ± 1.58
cya-3-glc [mg/L]	7.30 ± 1.33	7.37 ± 2.59	2.47 ± 2.36	2.43 ± 0.65	-	-
pet-3-glc [mg/L]	36.92 ± 3.63	36.58 ± 4.02	24.15 ± 3.86	19.72 ± 0.44	13.91 ± 1.50	12.75 ± 1.20
peo-3-glc [mg/L]	29.37 ± 2.68	29.79 ± 4.93	14.05 ± 1.82	11.37 ± 1.23	5.85 ± 0.81	5.59 ± 0.06
mal-3-glc [mg/L]	178.27 ± 7.64	177.30 ± 6.22	103.43 ± 2.86	89.05 ± 4.92	113.30 ± 5.19	105.72 ± 5.10
del-3-glac [mg/L]	11.01 ± 1.44	10.85 ± 1.64	8.53 ± 1.65	7.03 ± 0.49	4.18 ± 0.44	4.07 ± 0.15
pet-3-glac [mg/L]	8.98 ± 1.04	8.81 ± 1.11	7.50 ± 1.40	5.97 ± 0.64	4.68 ± 0.56	4.45 ± 0.53
peo-3-glac [mg/L]	5.63 ± 0.34	5.51 ± 0.52	3.98 ± 0.60	2.78 ± 0.45	2.03 ± 0.06	1.83 ± 0.21
mal-3-glac [mg/L]	41.87 ± 1.51	42.33 ± 1.11	29.55 ± 3.25	24.52 ± 1.14	40.11 ± 1.56	37.62 ± 4.32
pet-3-glcu [mg/L]	1.36 ± 0.21	1.19 ± 0.20	-	-	0.66 ± 0.14	0.47 ± 0.27
peo-3-glcu [mg/L]	2.32 ± 0.18	2.37 ± 0.25	-	-	0.74 ± 0.16	0.66 ± 0.04
mal-3-glcu [mg/L]	27.39 ± 1.10	26.67 ± 1.12	-	-	-	-
MI	33.50 ± 4.01	38.07 ± 3.41	28.87 ± 7.15	30.34 ± 4.16	28.77 ± 5.73	26.00 ± 2.77

Values are the mean (± SD) of three FACE rings per treatment with aCO_2_ (A1, A2 and A3) and eCO_2_ (E1, E2 and E3).

## Data Availability

Data sharing not applicable.

## Supplementary Materials

The following are available online at https://www.mdpi.com/2304-8158/10/1/145/s1, Figure S1, posterior predicted difference (median and 50% HDI) between vintages within treatment (eCO_2_ and aCO_2_) from Bayesian generalized linear mixed effects models on analyzed must parameters of Cabernet Sauvignon (CS) and Riesling (R); Figure S2, posterior predicted difference (median and 50% HDI) between vintages within treatment (eCO_2_ and aCO_2_) from Bayesian generalized linear mixed effects models on analyzed wine parameters of Cabernet Sauvignon (CS) and Riesling (R); Figure S3, posterior predicted difference (median and 50% HDI) between vintages within treatment (eCO_2_ and aCO_2_) from Bayesian generalized linear mixed effects models on L*, A* and b* calculations of Cabernet Sauvignon (CS) and Riesling (R); Figure S4, posterior predicted difference (median and 50% HDI) between vintages within treatment (eCO_2_ and aCO_2_) from Bayesian generalized linear mixed effects models on anthocyanins and monomeric index of Cabernet Sauvignon (CS).
