# Supplementary material for: Responses on Must and Wine Composition of Vitis vinifera L. cvs. Riesling and Cabernet Sauvignon under a Free Air CO2 Enrichment (FACE)
Source: Foods. 2021 Jan 12;10(1):145. doi: 10.3390/foods10010145 (PMC7828110; doi:10.3390/foods10010145)
Supplement: Supplementary file 1 [file foods-10-00145-s001.pdf]

foods

MDPI

Supplementary Material:

Responses on must and wine composition of *Vitis vinifera* L. cvs. Riesling and Cabernet Sauvignon under a Free Air CO_2 Enrichment (FACE)

Yvette Wohlfahrt [1,*], Claus-Dieter Patz [2], Dominik Schmidt [3], Doris Rauhut [4], Bernd Honermeier [5], Manfred Stoll [1]

[1]Hochschule Geisenheim University, Department of General and Organic Viticulture, 65366 Geisenheim, Germany
[2]Hochschule Geisenheim University, Department of Beverage Research, 65366 Geisenheim, Germany
[3]Hochschule Geisenheim University, Department of Modeling and Systems Analysis, 65366 Geisenheim, Germany
[4]Hochschule Geisenheim University, Department of Microbiology and Biochemistry, 65366 Geisenheim, Germany
[5]Justus Liebig University, Institute of Agronomy and Plant Breeding I, 35392 Giessen, Germany

* Corresponding author
E-mail: yvette.wohlfahrt@hs-gm.de; Phone: +49 6722 144

ORCID
Yvette Wohlfahrt: 0000-0002-2496-781X
Claus-Dieter Patz: 0000-0002-7080-1917
Dominik Schmidt: 0000-0001-9480-2001
Bernd Honermeier: 0000-0003-4709-2136

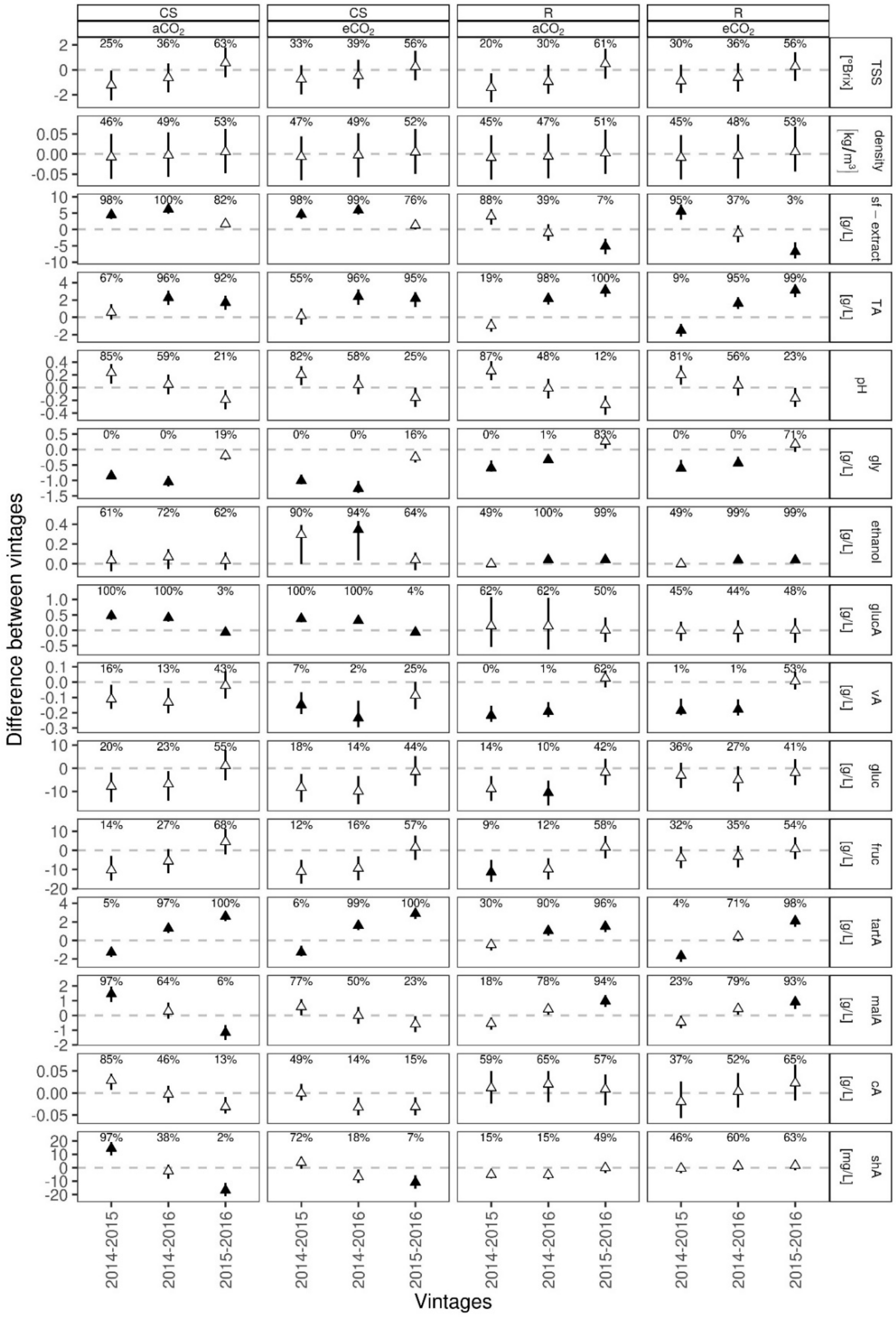

Figure S1. Posterior predicted difference (median and 50% HDI) between vintages within treatment (eCO_2 and aCO_2) from Bayesian generalized linear mixed effects models on analyzed must parameters of Cabernet Sauvignon (CS) and Riesling (R). Percentages represent the probability of Vintage1-Vintage2 > 0. Filled symbols indicate "significant differences", if the probability is >90% (positive difference) or <10% (negative difference).

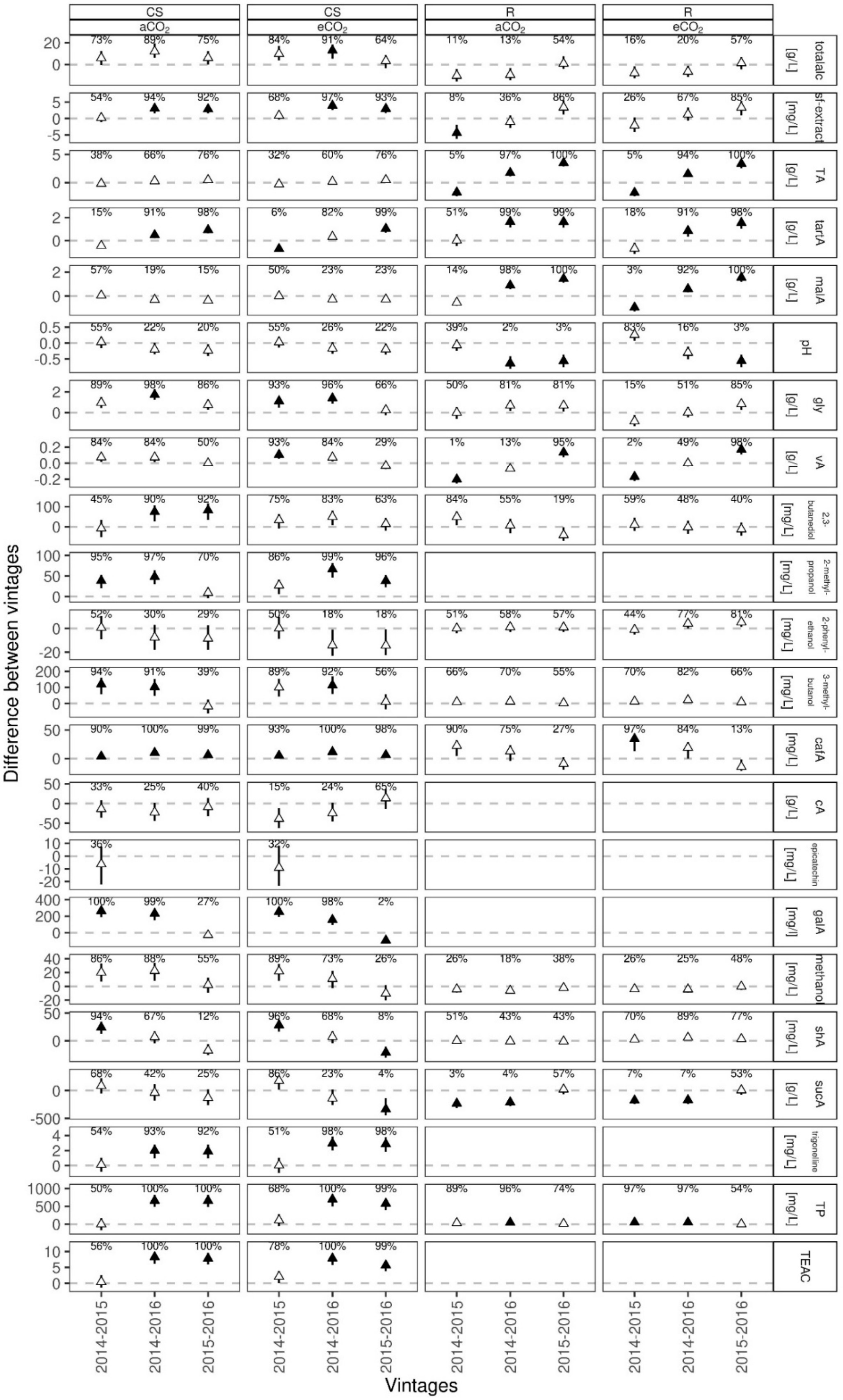

Figure S2. Posterior predicted difference (median and 50% HDI) between vintages within treatment (eCO_2 and aCO_2) from Bayesian generalized linear mixed effects models on analyzed wine parameters of Cabernet Sauvignon (CS) and Riesling (R). Percentages represent the probability of Vintage1-Vintage2 > 0. Filled symbols indicate "significant differences", if the probability is >90% (positive difference) or <10% (negative difference).

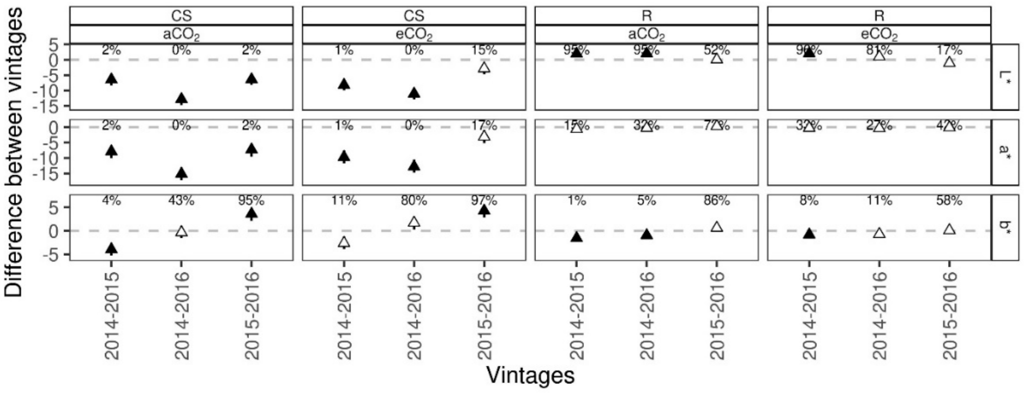

Figure S3. Posterior predicted difference (median and 50% HDI) between vintages within treatment (eCO_2 and aCO_2) from Bayesian generalized linear mixed effects models on L*, A* and b* calculations of Cabernet Sauvignon (CS) and Riesling (R). Percentages represent the probability of Vintage1-Vintage2 > 0. Filled symbols indicate "significant differences", if the probability is >90% (positive difference) or <10% (negative difference).

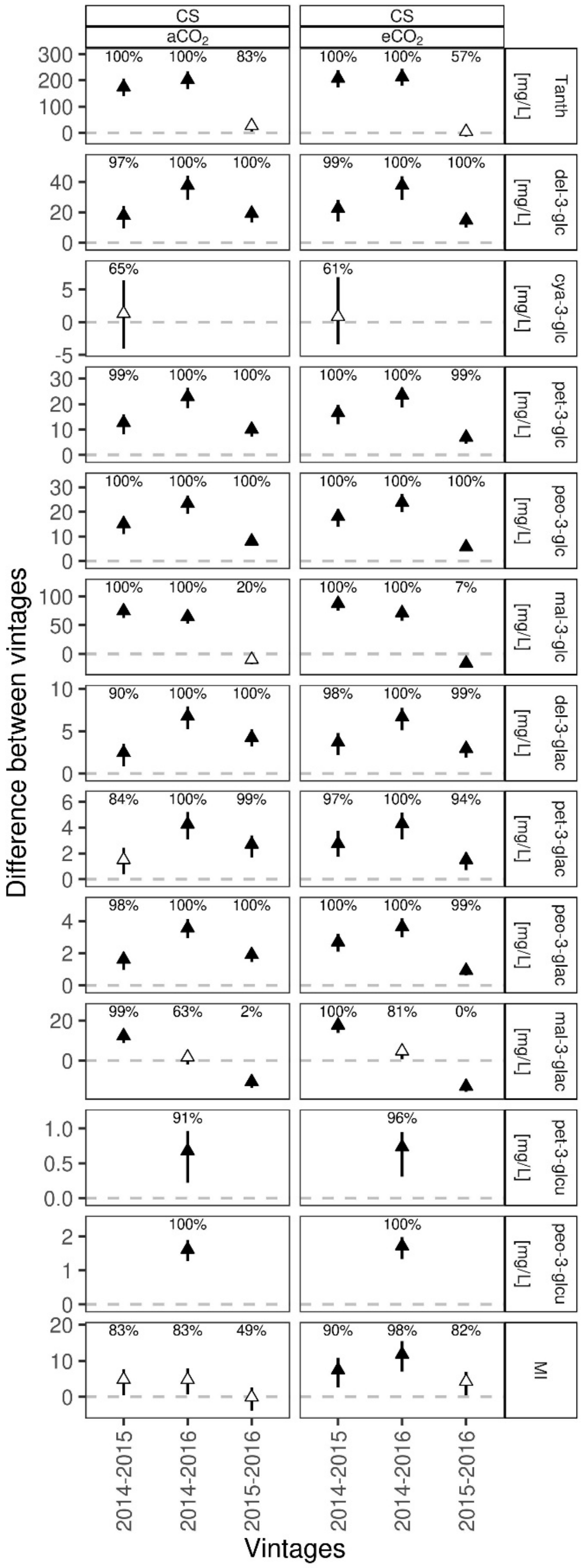

Figure S4. Posterior predicted difference (median and 50% HDI) between vintages within treatment (eCO_2 and aCO_2) from Bayesian generalized linear mixed effects models on anthocyanins and monomeric index of Cabernet Sauvignon (CS). Percentages represent the probability of Vintage1-Vintage2 > 0. Filled symbols indicate “significant differences”, if the probability is >90% (positive difference) or <10% (negative difference).
